# Complement regulator CD59 prevents peripheral organ injury in rats made seropositive for neuromyelitis optica immunoglobulin G

**DOI:** 10.1186/s40478-017-0462-4

**Published:** 2017-07-27

**Authors:** Xiaoming Yao, Alan S. Verkman

**Affiliations:** Departments of Medicine and Physiology, University of California, 1246 Health Sciences East Tower, San Francisco, CA 94143-0521 USA

**Keywords:** NMO, Aquaporin-4, Complement inhibitor, Skeletal muscle, Kidney, Astrocyte, Transgenic rat

## Abstract

Pathogenesis in aquaporin-4 immunoglobulin G (AQP4-IgG) seropositive neuromyelitis optica spectrum disorders (herein called NMO) involves complement-dependent cytotoxicity initiated by AQP4-IgG binding to astrocyte AQP4. We recently reported that rats lacking complement inhibitor protein CD59 were highly susceptible to development of NMO pathology in brain and spinal cord following direct AQP4-IgG administration (Yao and Verkman, *Acta Neuropath Commun* 2017, 5:15). Here, we report evidence that CD59 is responsible for protection of peripheral, AQP4-expressing tissues in seropositive NMO. Rats made seropositive by intraperitoneal injection of AQP4-IgG developed marked weakness by 24 h and died soon thereafter. Serum creatine phosphokinase at 24 h was >900-fold greater in seropositive CD59^−/−^ rats than in seropositive CD59^+/+^ (or control) rats. AQP4-expressing cells in skeletal muscle and kidney, but not in stomach, of seropositive CD59^−/−^ rats showed injury with deposition of AQP4-IgG and activated complement C5b-9, and inflammation. Organ injury in seropositive CD59^−/−^ rats was prevented by a complement inhibitor. Significant pathological changes in seropositive CD59^−/−^ rats were not seen in optic nerve, spinal cord or brain, including circumventricular tissue. These results implicate a major protective role of CD59 outside of the central nervous system in seropositive NMO, and hence offer an explanation as to why peripheral, AQP4-expressing cells are largely unaffected in NMO.

**Electronic supplementary material:**

The online version of this article (doi:10.1186/s40478-017-0462-4) contains supplementary material, which is available to authorized users.

## Introduction

Complement-mediated cytotoxicity plays a central role in the pathogenesis of seropositive neuromyelitis optica spectrum disorders (herein called NMO), in which immunoglobulin G autoantibodies against water channel aquaporin-4 (AQP4), called AQP4-IgG, bind to astrocytes in brain, spinal cord and optic nerve [9, 10]. Following astrocyte injury, downstream inflammation, blood-brain barrier disruption and potentially additional mechanisms result in oligodendrocyte injury and demyelination [11, 17, 29]. Optic neuritis and transverse myelitis are the major clinical manifestations of NMO, which can produce visual and motor impairment, and brain involvement can occur as well. Though AQP4 is expressed in some peripheral organs outside of the central nervous system, including skeletal muscle, kidney and stomach [8, 19], these organs are rarely affected in NMO, with only a few reports of NMO-associated myositis associated with elevated creatine phosphokinase and skeletal muscle pathology [7, 13, 27].

It has been unclear why peripheral AQP4-expressing cells, which are exposed to high levels of circulating AQP4-IgG in seropositive NMO, are largely spared. Rodent studies show prompt deposition of circulating AQP4-IgG on peripheral AQP4-expressing cells [1, 20], and humans can be AQP4-IgG seropositive for many years prior to clinical manifestations of NMO [16]. Rodents administered AQP4-IgG systemically do not spontaneously develop pathological changes in the central nervous system or in peripheral organs, though pathology in brain is seen following mechanical disruption of the blood-brain barrier [1].

Here, we tested the hypothesis that complement regulator protein CD59 is responsible for protection of AQP4-expressing peripheral cells in seropositive NMO. CD59 is a phosphoinositol-linked membrane glycoprotein that inhibits formation of the complement terminal membrane attack complex [6, 21, 26, 34]. The motivation for this work is the expression of CD59 in AQP4-expressing cells in the central nervous system and in the periphery, and our prior data showing that CD59^−/−^ mice are highly sensitive to development of NMO pathology following direct administration of AQP4-IgG and human complement into brain or cerebrospinal fluid [33]. CD59^−/−^ rats also develop marked brain and spinal cord pathology following direct AQP4-IgG administration [32], albeit without the need for coadministration of complement, because rats, unlike mice, have an active classical complement system [2, 22]. We report here marked injury to skeletal muscle following systemic AQP4-IgG administration to CD59^−/−^ rats, offering an explanation for the sparing of peripheral organs in seropositive NMO.

## Materials and methods

### Materials

Purified recombinant AQP4-IgG (rAb-53) [3, 5] was provided by Dr. Jeffrey Bennett (Univ. Colorado, Denver) and (non-NMO) pooled human IgG, as control, was purchased from Pierce Biotechnology (Rockford, IL). Some studies were done using an antibody fragment-based complement inhibitor, herein called Comp_inh_ (to be reported separately), which inhibits serum complement activity by >95% for 8–12 h after intravenous administration. Unless otherwise specified chemicals were purchased from Sigma-Aldrich (St. Louis, MO).

### CD59^−/−^ rats

CD59^−/−^ rats in a Sprague-Dawley background were custom-generated by Transposagen Biopharm. Inc. (Lexingtobon, KY) using CRISPR-Cas9 gene targeting technology as described [32]. In vivo studies were done on 8- to 10-week-old, weight-matched CD59^+/+^ and CD59^−/−^ rats. Rats were maintained in air-filtered cages and fed normal rat chow in the University of California, San Francisco (UCSF) Animal Care facility. All procedures were approved by the UCSF Committee on Animal Research (approval number AN108551).

### Blood and urine analysis

Blood (800 μL) was collected before and 24 h after AQP4-IgG injection. Hematological parameters were measured using a Genesis Hematology Analyzer (Oxford Science, Oxford, CT) in which 80 μL blood was collected into a MiniCollect tube with EDTA (Greiner Bio-One GmbH, Kremsmunster, Austria). Serum was obtained by collection of blood in 1.5-ml Eppendoff tubes then allowing clotting for 30 min followed by centrifugation at 800 g for 10 min at 4 °C. Serum was frozen at −70 °C for blood chemistry analyses (IDEXX BioResearch, Sacramento, CA). Urine was collected for determination of osmolality using a freezing-point depression osmometer (Model 3320 Osmometer, Advanced Instruments Inc., Natick, MA).

### AQP4-IgG pharmacokinetics

Adult CD59^+/+^ and CD59^−/−^ rats were administered AQP4-IgG (4 mg/kg body weight in PBS) by intraperitoneal injection in a total volume of 500 μL. Blood was collected through the tail vein at 1, 2, 4, 6, 8, 24 and 48 h, left for 30 min at room temperature to allow clotting, and centrifuged for 10 min at 800 g at 4 °C. Serum was diluted 50,000-fold and human IgG concentration was determined using a human IgG ELISA kit (GenWay, San Diego, CA).

### Intraperitoneal injection model

AQP4-IgG or (non-NMO) control human IgG (5 mg/kg body weight) was injected intraperitoneally. At 24 h, clinical motor scores were recorded, as adapted from scoring used for neuroinflammation models in rodents [18]: score 0, normal movement; score 1, tail paralysis; score 2, hindlimb paralysis; score 3, hindlimb and frontlimb paresis with breathing difficulty; score 4, complete paralysis with moribund condition. Then rats were deeply anesthetized using ketamine (100 mg/kg) and xylazine (10 mg/kg) and transcardially perfused with 200 mL heparinized PBS and 200 mL of 4% PFA in PBS. Brain, optic nerve, spinal cord (cervical), skeletal muscle, kidney and stomach were removed and post-fixed for 4 h in 4% paraformaldehyde (PFA) and cryoprotected in 20% sucrose. Tissues were embedding in OCT compound and sectioned at 7-μm thickness using a cryostat (CM1900, Leica) for immunofluorescence. For complement inhibition studies, Comp_inh_ (50 mg/kg) was injected intravenously just before and 12 h after intraperitoneal AQP4-IgG (or control IgG) administration.

### Immunofluorescence

Frozen sections of harvested tissues were immunostained as described [32]. Briefly, sections were incubated in blocking solution (1% BSA containing 0.3% Triton X-100 in PBS) for 1 h at room temperature, then incubated overnight at 4 °C with primary antibodies against AQP4 (1:200, Santa Cruz Biotechnology), human IgG (1:200, Santa Cruz Biotechnology), C5b-9 (1:100, Hycult Biotech, PA), CD45 (1:50, Cambridge, MA), CD59 and CD55 (1:50, LSBio, Seattle, WA), CD46 (1:50, Abcam, MA), GFAP (1:200; Millipore), ionized calcium binding adaptor molecule 1 (Iba1; 1:400, Wako, Richmond, VA), or myelin basic protein (MBP, 1:100, Santa Cruz Biotechnology), followed by the appropriate species-specific Alexa Fluor-conjugated secondary antibody for 1 h (5 μg/mL each, Invitrogen) at room temperature. Sections were mounted with Prolong Gold antifade reagent with DAPI (Invitrogen, Life Technologies, Eugene, OR) for visualization of immunofluorescence on a Leica fluorescence microscope or Nikon confocal microscope.

### Statistics

Data are presented as mean ± S.E.M. Statistical analysis was performed using Prism 5 GraphPad Software package (San Diego, CA). The normality of the data was established by Bartlett’s test for equal variances and a one-way ANOVA with Newmann-Keuls post-hoc test to compare groups.

## Results

### Expression of complement inhibitor proteins in AQP4-expressing organs

**Fig. 1:**
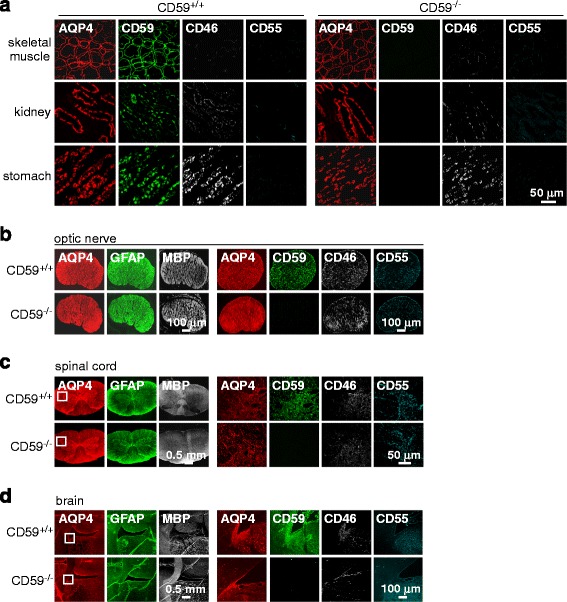
Expression of AQP4 and complement regulator proteins CD59, CD55, and CD46 in peripheral organs and central nervous system. Immunofluorescence in skeletal muscle, kidney and stomach (**a**), optic nerve (**b**), spinal cord (**c**), and brain (in circumventricular region) (**d**). Micrographs in c and d shown at low and high (boxed region) magnifications. Representative of studies done in 3 rats per genotype

Immunofluorescence of AQP4 and complement inhibitor proteins in Fig. 1a shows coexpression of AQP4 and CD59 in three major peripheral tissues in which AQP4 is expressed – skeletal muscle, kidney and stomach. Staining was seen in sarcolemma in skeletal muscle, in basolateral plasma membrane in inner medullary collecting duct in kidney, and in parietal cells in glands in stomach. CD59 immunofluorescence was absent in organs from CD59^−/−^ rats, and AQP4 immunofluorescence did not differ significantly in tissues from CD59^+/+^ and CD59^−/−^ rats (relative to 1.0 for CD59^+/+^ rats: 1.01 ± 0.07 (skeletal muscle); 1.04 ± 0.07 (kidney); 0.96 ± 0.09 (stomach)). Complement inhibitor protein CD46 was seen in stomach > kidney > skeletal muscle, with similar expression in CD59^+/+^ and CD59^−/−^ rats. Relatively little CD55 was seen in these peripheral organs.

Immunofluorescence in optic nerve, spinal cord and brain showed gross colocalization of astrocyte markers AQP4 and GFAP, with a distinct pattern of myelin (MBP) expression (Fig. 1b–d, left 3 panels). AQP4 coexpression with CD59 was seen in the three CNS tissues, with strongest CD59 immunofluorescence in brain, with different levels of CD46 and CD55 seen in the various tissues (right 4 panels). AQP4 immunofluorescence did not differ significantly in tissues from CD59^+/+^ and CD59^−/−^ rats (relative to 1.0 for CD59^+/+^ rats: 1.01 ± 0.06 (optic nerve); 0.97 ± 0.04 (spinal cord); 0.96 ± 0.09 (brain)). CD59 immunofluorescence was absent in CNS tissues from CD59^−/−^ rats, and CD46 and CD55 immunofluorescence was similar in CD59^+/+^ and CD59^−/−^ rats.

### Marked weakness and serum CK elevation in CD59^−/−^ rats following systemic AQP4-IgG administration

**Fig. 2:**
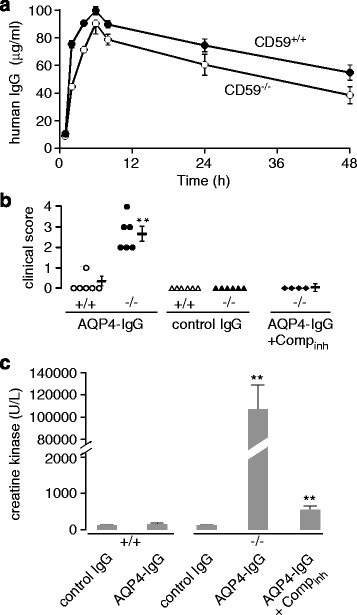
Marked weakness and CK elevation in CD59^−/−^ rats following systemic AQP4-IgG administration. **a** AQP4-IgG pharmacokinetics in CD59^+/+^ and CD59^−/−^ rats (mean ± S.E.M., 3 rats per genotype). **b** Clinical motor score at 24 h following IP injection of AQP4-IgG. Data shown for individual rats along with mean ± S.E.M. **c** Serum CK for indicated groups (mean ± S.E.M., 6 rats per genotype, except 4 rats for Comp_inh_-treated group, **P < 0.01 comparing to AQP4-IgG-treated AQP4^+/+^ rats)

AQP4-IgG pharmacokinetics in CD59^+/+^ and CD59^−/−^ rats was measured following a single intraperitoneal injection of AQP4-IgG. Human IgG concentration in rat serum was assayed by ELISA against human IgG, which does not detect rat IgG. Fig. 2a shows human IgG concentration in rat serum over 48 h. AQP4-IgG concentration increased over the first few hours as it was absorbed from the peritoneal cavity, was maximum at ~6 h, and then decreased with t_1/2_ ~ 48 h. Slightly lower AQP4-IgG concentrations were seen in CD59^−/−^ compared to CD59^+/+^ rats.

All AQP4-IgG-treated CD59^−/−^ rats developed marked weakness and motor disability at 24 h, as exemplified in the short clip in Additional file 1 Video S1, with motor score data summarized in Fig. 2b. Little or no motor disability was seen in AQP4-IgG-treated CD59^+/+^ rats, in control (non-NMO) human IgG-treated CD59^+/+^ and CD59^−/−^ rats, and in AQP4-IgG-treated CD59^−/−^ rats administered a complement inhibitor just before and 12 h after AQP4-IgG administration. There was remarkable, >900-fold elevation in serum CK in the AQP4-IgG treated CD59^−/−^ rats, which was largely prevented by complement inhibition (Fig. 2c). Though there are several potential sources of CK, the exceptionally high levels found in the treated rats are very likely to come from skeletal muscle.

Tables 1 and 2 summarize hematological parameters and chemistries of control and AQP4-IgG-treated rats. The data indicate a mild hemolytic anemia in untreated CD59^−/−^ rats as evidenced by reduced hematocrit and mild reticulocytosis, as has been reported in humans lacking CD59 [4, 15]. In AQP4-IgG-treated CD59^−/−^ rats, in addition to the marked elevation in CK, changes were seen in liver enzymes (AST and ALT) and kidney indices (BUN and creatinine), which is likely due to secondary effects of muscle injury, rhabdomyolysis and dehydration with reduced food intake. The treated CD59^−/−^ rats also manifested a urinary concentrating defect (urine osmolality: AQP4-IgG-treated 521 ± 96 mOsm vs. control IgG-treated 1413 ± 155 mOsm, S.E.M. 6 rats per group). The hemoconcentration in CD59^−/−^ rats is likely due to reduced fluid intake and dehydration.

**Table 1:** Hematological parameters before and 24 h after IP injection of AQP4-IgG in CD59^−/−^ and CD59^+/+^ rats

	Hb (g/dl)	RBC (1012/L)	HCT (%)	RDW (%)	Retic (%)	WBC (109/L)	Plt (109/L)
Before AQP4-IgG administration							
CD59^+/+^	15.1 ± 2.0	9.2 ± 1.2	44.4 ± 6.0	13.5 ± 1.9	0.0 ± 0.0	7.5 ± 0.7	870 ± 162
CD59^−/−^	13.9 ± 1.1	8.5 ± 0.9	41.5 ± 4.4	13.4 ± 1.0	0.4 ± 0.2^+^	8.1 ± 1.4	828 ± 114
24 h after AQP4-IgG administration							
CD59^+/+^	13.6 ± 1.1	8.8 ± 0.9	40.8 ± 3.0	13.3 ± 1.9	0.0 ± 0.0	6.8 ± 3.4	741 ± 207
CD59^−/−^	18.4 ± 1.8*	11.4 ± 1.9*	55.7 ± 6.1*	13.6 ± 0.9	0.3 ± 0.1^+^	9.1 ± 5.6	685 ± 195
CD59^−/−^ + Comp_inh_	13.4 ± 0.5	7.6 ± 0.2	40.0 ± 2.4	12.6 ± 0.7	0.3 ± 0.2^+^	5.2 ± 1.2	686 ± 99

Mean ± S.E.M. of 4–6 rats per genotype (three males and three females)

*Hb* hemoglobin, *RBC* red blood cell count, *HCT* hematocrit, *RDW* RBC distribution width; *Retic* reticulocyte count, *WBC* white blood cell count, *Plts* platelet count

*p < 0.01 comparing with AQP4-IgG-treated CD59^+/+^ rats. ^+^ *p* < 0.01 comparing with untreated CD59^+/+^ rats

**Table 2:** Blood chemistries before and 24 h after IP injection of AQP4-IgG in CD59^−/−^ and CD59^+/+^ rats

Parameters	CD59^+/+^ before	CD59^+/+^ after	CD59^−/−^ before	CD59^−/−^ after	CD59^−/−^ +Comp_inh_
ALP (U/L)	106 ± 77	63 ± 60	142 ± 93	117 ± 89	122 ± 21
AST (U/L)	60 ± 9	81 ± 23	129 ± 51	5784 ± 1021*	252 ± 34*
ALT (U/L)	36 ± 9	30 ± 9	50 ± 10	1182 ± 298*	60 ± 14
Creatine Kinase (U/L)	85 ± 9	120 ± 51	92 ± 9	106,767 ± 42901*	547 ± 84*
Albumin (g/dL)	2.9 ± 0.2	2.9 ± 0.3	2.9 ± 0.2	2.9 ± 0.3	2.8 ± 0.3
Total Bilirubin (mg/dL)	0.1 ± 0.1	0.1 ± 0.0	0.1 ± 0.0	0.2 ± 0.1	0.1 ± 0.0
Total Protein (g/dL)	5.8 ± 0.3	6.1 ± 0.3	5.8 ± 0.3	6.7 ± 0.6	5.6 ± 0.3
Globulin (g/dL)	2.9 ± 0.4	3.2 ± 0.4	2.9 ± 0.2	3.8 ± 0.1	2.8 ± 0.1
BUN (mg/dL)	13.6 ± 2.9	13.3 ± 3.3	14.7 ± 1.3	124 ± 46*	16.0 ± 4.4
Creatinine (mg/dL)	0.2 ± 0.0	0.3 ± 0.1	0.2 ± 0.1	1.2 ± 0.8*	0.3 ± 0.1
Cholesterol (mg/dL)	84 ± 20	83 ± 21	69 ± 11	110 ± 25	71 ± 10
Glucose (mg/dL)	208 ± 32	183 ± 14	198 ± 39	148 ± 82	205 ± 12
Calcium (mg/dL)	6.9 ± 4.4	5.1 ± 4.9	7.1 ± 4.2	6.2 ± 3.9	9.9 ± 0.2
Phosphorus (mg/dL)	5.3 ± 0.8	6.1 ± 1.1	6.0 ± 0.9	16.7 ± 6.1	6.9 ± 0.7
Bicarbonate (mmol/L)	20 ± 4	21 ± 2	21 ± 2	20 ± 4	22 ± 2
Chloride (mmol/L)	99 ± 4	98 ± 5	100 ± 3	90 ± 5	105 ± 3
Sodium (mmol/L)	136 ± 8	140 ± 2	138 ± 5	136 ± 6	143 ± 3

Mean ± S.E.M. of 4–6 rats per genotype (three males and three females)

*ALP* alkaline phosphatase, *AST/ALT* aspartate aminotransferase/alanine aminotransferase, *CK* creatine kinase, *BUN* blood urea nitrogen

*p < 0.01 comparing with untreated CD59^−/−^ rats

### Pathology in peripheral, AQP4-expressing organs in AQP4-IgG seropositive CD59^−/−^ rats

**Fig. 3:**
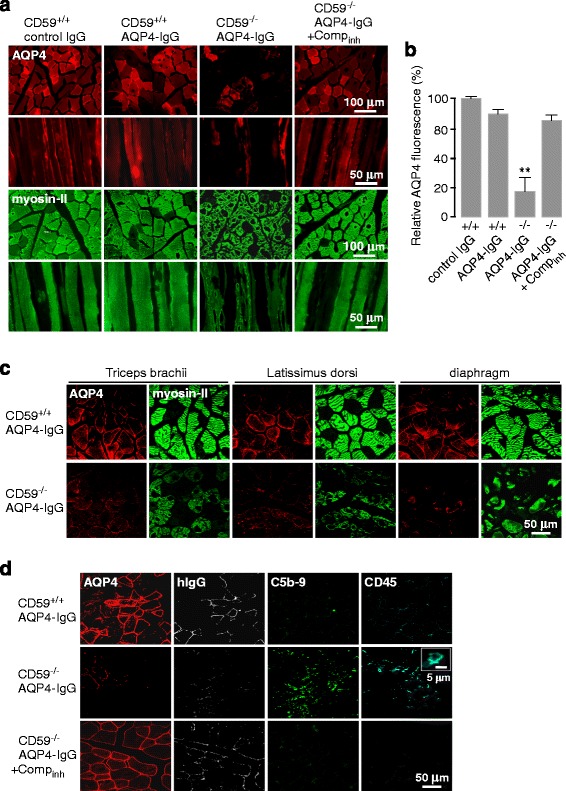
Immunofluorescence in skeletal muscle at 24 h after intraperitoneal AQP4-IgG administration. **a** AQP4 and myosin-II immunofluorescence of tibialis anterior muscle, representative of 4–6 rats per group. **b** Relative AQP4 immunofluorescence (mean ± S.E.M., 4–6 rats per group, ** *P* < 0.01 compared to control IgG group). **c** Immunofluorescence of front limb (triceps brachii), back (latissimus dorsi) and diaphragm muscle in AQP4-IgG-treated CD59^+/+^ and CD59^−/−^ rats as in panel a, representative of 3 rats. **d** AQP4, hIgG, C5b-9 and CD45 immunofluorescence in tibialis anterior muscle. Representative of 3 rats per group

AQP4 and myosin-II immunofluorescence in hindlimb (tibialis anterior) muscle showed marked injury in AQP4-IgG treated CD59^−/−^ rats, with vacuole formation and disorganized myofibrils (Fig. 3a). Little or no abnormalities were seen in control (non-NMO) human IgG-treated CD59^+/+^ rats, AQP4-IgG-treated CD59^+/+^ rats, or complement inhibitor / AQP4-IgG-treated CD59^−/−^ rats. AQP4 immunofluorescence showed a patchy expression pattern and was significantly reduced in skeletal muscle of AQP4-IgG-treated CD59^−/−^ rats, with quantitative data summarized in Fig. 3b. Similar pathology was seen in skeletal muscle from forelimb (triceps brachii), back (latissimus dorsi) and diaphragm (Fig. 3c). Fig. 3d shows deposition of activated complement (C5b-9) and inflammatory cell infiltration (CD45) in tibialis anterior muscle of AQP4-IgG-treated CD59^−/−^ rats. We did not stain for leukocyte subtypes. AQP4-IgG deposition (hIgG staining) was seen in the AQP4-IgG-treated CD59^+/+^ rats, but to a lesser extent in CD59^−/−^ rats where AQP4 was largely gone. CD59 thus plays an important role in protection of skeletal muscle in seropositive NMO.

**Fig. 4:**
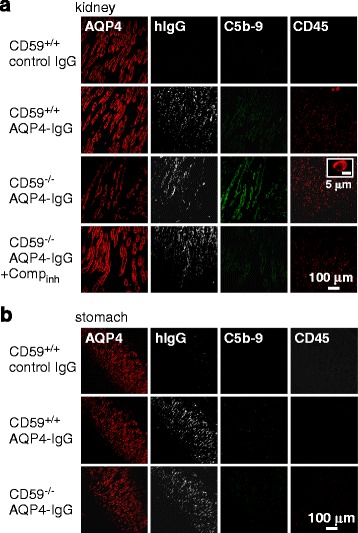
Immunofluorescence in kidney and stomach at 24 h after intraperitoneal AQP4-IgG administration. AQP4, hIgG, C5b-9 and CD45 immunofluorescence in kidney (**a**) and stomach (**b**). Representative of 3 rats per group

In kidney inner medulla where AQP4-expressing inner medullary collecting ducts are located, AQP4-IgG-treated rats showed loss of AQP4 immunofluorescence, as well as some deposition of activated complement and inflammatory cell infiltration, each of which were prevented by complement inhibition (Fig. 4a). Interestingly, in stomach, no significant changes in AQP4 expression were seen in AQP4-IgG-treated CD59^−/−^ rats, nor was there demonstrable deposition of activated complement or inflammatory cell infiltration (Fig. 4b).

### Absence of pathology in the central nervous system of AQP4-IgG seropositive CD59^−/−^ rats

**Fig. 5:**
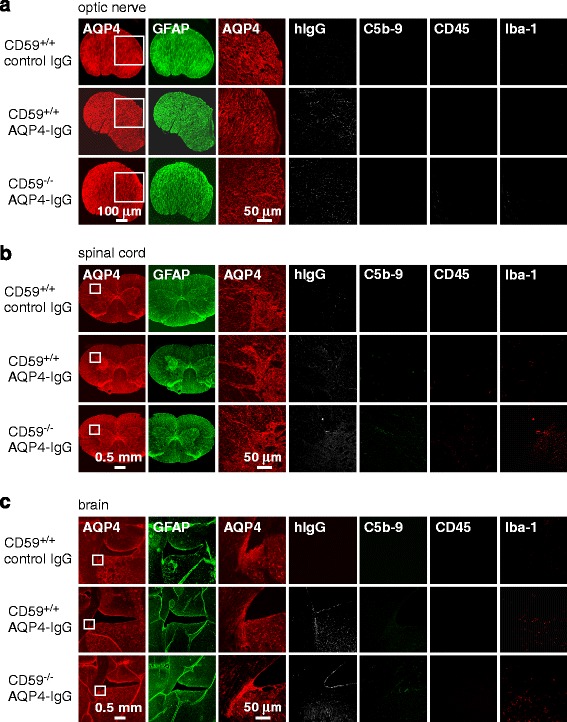
Immunofluorescence in optic nerve, spinal cord, and brain at 24 h after intraperitoneal AQP4-IgG administration. AQP4 (low and high magnification of boxed area), GFAP, hIgG, C5b-9, CD45 and Iba-1 immunofluoresence of optic nerve (**a**), spinal cord (**b**) and periventricular brain (**c**). Representative of 3 rats per group

Examination of optic nerve (Fig. 5a), spinal cord (Fig. 5b) and circumventricular brain (Fig. 5c) did not show NMO pathology in AQP4-IgG-treated CD59^−/−^ rats. AQP4 expression was similar to that in control CD59^+/+^ rats, and neither complement deposition nor inflammation (CD45 and Iba-1) was seen. AQP4-IgG deposition (hIgG) was not seen in optic nerve or spinal cord, suggesting that AQP4-IgG cannot access these tissues over the 24-h time. hIgG staining was, however, mildly positive in circumventricular brain tissue that lacks a tight blood-brain barrier.

## Discussion

The principal finding here is that rats lacking complement inhibitor protein CD59 develop marked weakness and pathological changes in AQP4-expressing skeletal muscle following systemic administration of AQP4-IgG, whereas under identical conditions wildtype rats do not. Mild pathological changes were also seen in AQP4-expressing epithelial cells in the renal inner medullary collecting duct, but not in AQP4-expressing gastric parietal cells. Injured AQP4-expressing cells in skeletal muscle of CD59^−/−^ rats showed reduced AQP4 expression, deposition of activated complement, and inflammation. Skeletal muscle injury was associated with marked elevation in serum creatine phosphokinase, which was largely prevented by complement inhibition, supporting the conclusion that complement-dependent cytotoxicity is responsible for peripheral organ injury in the seropositive CD59^−/−^ rats. The absence of demonstrable brain or spinal cord injury suggests that the marked motor dysfunction seen by 24 h is the consequence of acute skeletal muscle injury rather than central nervous system injury. Respiratory failure because of diaphragmatic involvement may have contributed to the early mortality. The study here required CD59^−/−^ rats rather than mice because of the low activity of mouse complement, precluding the use of mice to study the consequences of systemic AQP4-IgG seropositivity.

There are a few reports of NMO myositis with elevated serum CK, though no reports of NMO-associated pathology in other AQP4-expressing peripheral organs. In a report of 2 seropositive NMO patients with diffuse myalgias, a substantial transient elevation of CK was found, with muscle biopsy showing reduced AQP4 expression and deposition of activated complement [13], similar to the pathological changes seen in seropositive CD59^−/−^ rats here. One case of CK elevation at the time of NMO attacks was reported, but without muscle findings [7]. In a retrospective study of 733 cases of NMO in Japan, three patients were found with CK elevation and general fatigue weeks before symptoms of optic neuritis [27]. Though renal disease has not been reported in NMO, perhaps some patients manifest a mild urinary concentrating defect, as found in AQP4 knockout mice [12], and perhaps urinalysis might reveal cellular evidence of collecting duct injury.

Our findings support the conclusion that peripheral organ injury is largely absent in seropositive NMO because of protection by CD59 against complement-mediated injury in AQP4-expressing cells. Perhaps the myositis seen in a very small number of NMO patients is caused by altered CD59 expression or CD59 polymorphisms, though this possibility has not been investigated. It would also be interesting to study the expression of CD59 and other complement regulator proteins in central nervous system and peripheral organ in human NMO. The apparent greater sensitivity of skeletal muscle to complement-induced injury, compared with other AQP4-expressing peripheral organs, might be related to the greater metabolic activity of skeletal muscle cells, or perhaps to differences in the expression of other complement inhibitors. Our results also support the central role of CD59 in the modulation of AQP4-IgG-initiated complement injury and hence suggest the potential therapeutic benefit in NMO of upregulation of astrocyte CD59 or alternative complement regulator proteins by pharmacological or other means.

Previously, various explanations have been offered for the general absence of NMO disease in peripheral, AQP4-expressing organs. Once set of explanations focus on AQP4, postulating that differences in AQP4 expression, localization or membrane clustering affect AQP4-IgG binding and consequent complement activation [14, 25]. However, the robust binding of AQP4-IgG to peripheral AQP4-expressing cells in rodents argues strongly against an AQP4-centric explanation, as do AQP4 biochemical and freeze-fracture electron microscopy studies [19, 23, 30, 31]. AQP4-IgG is deposited in skeletal muscle where it has been studied in seropositive humans [13], and AQP4-IgG is rapidly deposited in multiple AQP4-expressing peripheral organs following its systemic delivery to rodents [1, 20]. Another possible explanation for sparing of peripheral organs in NMO is the unique milieu of the central nervous system, which, unlike peripheral organs, might amplify an inflammatory response because of the presence of microglia, a narrow extracellular space, and a blood-brain barrier that impedes inflammatory cell exit. However, there is no experimental evidence to support this explanation.

The findings here raise the question of why CD59 in astrocytes does not fully protect against AQP4-IgG/complement injury as it appears to do in peripheral organs. Our prior data using mouse and rat models of NMO produced by injection of AQP4-IgG into brain or the spinal cerebrospinal space implicate a protective role for CD59 in brain and spinal cord in vivo [32, 33], which was supported by in vitro data in astrocyte cultures from knockout mice and rats or following CD59 enzymatic neutralization. Saadoun and Papadopoulos [24] reported that complement inhibitors, including CD59, are not protective against complement injury in CNS tissues. Though this conclusion is not supported by our findings in CD59^−/−^ rats and mice, they reported interesting immunofluorescence in mouse brain in which CD59 expression was seen on astrocyte cell bodies but not on AQP4-rich foot-processes near microvessels. We believe that higher-resolution imaging studies using electron microscopy or super-resolution fluorescence microscopy are needed to resolve unambiguously the subcellular localization of CD59 and AQP4 in brain sections. With regard to why CD59 is not fully protective against AQP4-IgG injury in the central nervous system, perhaps, as mentioned above, the unique cellular and physical milieu in brain, spinal cord and optic nerve, such as the presence of microglia and a narrow extracellular space, might amplify subthreshold AQP4-IgG-induced injury. We recently reported evidence for complement bystander injury to oligodendrocytes, which lack CD59, following exposure of nearby astrocytes to AQP4-IgG and complement [28]. While bystander cytotoxicity may be a major mechanism of cellular injury in the central nervous system, it may be inconsequential in peripheral AQP4-expressing organs.

Lastly, we acknowledge limitations in extrapolating the conclusions here from studies done in CD59^−/−^ rats to human NMO. Though the major anatomical structures are similar in rats and humans, there are differences in the ratios of various cell types in the central nervous system such as astrocytes and neurons, and there may be differences in expression levels of CD59 and other complement inhibitor proteins. Though there is compelling evidence for the pathogenicity of AQP4-IgG and complement, the passive-transfer model used here may not fully recapitulate the pathogenesis of seropositive NMO in humans, where additional mechanisms, perhaps cytotoxic T cells, might also contribute. Finally, the data here were from short-term follow-up after systemic AQP4-IgG administration to rats, which was necessitated by the severe pathology in AQP4-IgG-treated CD59^−/−^ rats. Peripheral organs in humans with seropositive NMO can be exposed to AQP4-IgG continuously for many years or decades. Notwithstanding these potential limitations, our results offer a logical explanation for the general absence of NMO disease outside of the central nervous system.

## Conclusions

Our results provide evidence that CD59 expression in peripheral, AQP4-expressing organs is responsible for the absence of peripheral organ injury in seropositive NMO.

## Additional file

Additional file 1: Video S1. AQP4^+/+^ rat (right) and AQP4^−/−^ rat (left) at 24 h after intraperitoneal AQP4-IgG administration. (MOV 2356 kb)
